# Regulation of fatty acid composition and lipid storage by thyroid hormone in mouse liver

**DOI:** 10.1186/2045-3701-4-38

**Published:** 2014-07-30

**Authors:** Xuan Yao, Sarina Hou, Duo Zhang, Hongfeng Xia, Yu-Cheng Wang, Jingjing Jiang, Huiyong Yin, Hao Ying

**Affiliations:** 1 Key Laboratory of Food Safety Research, Institute for Nutritional Sciences, Shanghai Institutes for Biological Sciences, Graduate School of the Chinese Academy of Sciences, Chinese Academy of Sciences, Shanghai 200031, China; 2 Clinical Research Center of Institute for Nutritional Sciences, Shanghai Institutes for Biological Sciences, Chinese Academy of Sciences, Shanghai 200031, China; 3 Department of Nutrition, Shanghai Xuhui Central Hospital, Shanghai 200031, China; 4 Department of Endocrinology and Metabolism, Zhongshan Hospital, Fudan University, Shanghai, China; 5 Key Laboratory of Food Safety Risk Assessment, Ministry of Health, Beijing, China

**Keywords:** Thyroid hormone, Liver, Fatty acid, Glycogen, NAFLD

## Abstract

**Background:**

Thyroid hormones (THs) are potent hormones modulating liver lipid homeostasis. The perturbation of lipid homeostasis is a hallmark of non-alcoholic fatty liver disease (NAFLD), a very common liver disorder. It was reported that NAFLD patients were associated with higher incidence of hypothyroidism. However, whether abnormal thyroid function contributes to the pathogenesis of NAFLD remains unclear.

**Results:**

We used *in vivo* models to investigate the influence of hypothyroidism and TH on hepatic lipid homeostasis. We did not observe hepatic triglyceride accumulation in the liver of hypothyroid mice, although the liver was enlarged. We then characterized the hepatic fatty acid composition with gas chromatography–mass spectrometry in mice under different thyroid states. We found that hypothyroidism decreased saturated fatty acid (SFA) content while TH treatment restored the level of SFA. In agreement with this finding, we observed that the expression of acetyl-CoA carboxylase 1 and fatty acid synthase, the rate-limit enzymes for de novo lipogenesis (DNL), decreased in hypothyroid mice while increased after TH treatment. We also found that the ratio of C18:1n-9/C18:0 and C16:1n-7/C16:0 was decreased by TH treatment, suggesting the activity of stearoyl-CoA desaturase-1 was suppressed. This finding indicated that TH is able to suppress triglyceride accumulation by reducing fatty acid desaturation. Additionally, we found that hepatic glycogen content was substantially influenced by TH status, which was associated with glycogen synthase expression. The increased glycogen storage might explain the enlarged liver we observed in hypothyroid mice.

**Conclusions:**

Taken together, our study here suggested that hypothyroidism in mice might not lead to the development of NAFLD although the liver became enlarged. However, disturbed TH levels led to altered hepatic fatty acid composition and glycogen accumulation.

## Background

Thyroid hormone (TH) is potent to influence multiple aspects of lipid, carbohydrate, protein and mineral metabolism [1]. Through binding to nuclear TH receptors (TR), TH can modulate the expression of target genes [2]. Physiological inverse relationship between TH, such as thyroxine (T4) and triiodothyronine (T3), and thyroid-stimulating hormone (TSH) are maintained through a classic negative feedback loop mediated by the hypothalamic-pituitary-thyroid axis. When the components of the feedback loop cannot act co-ordinately, it usually indicates incident of diseases. Overt hyperthyroidism is used to describe the situation when patients are found to have an undetectable TSH level and a high T4 or T3 level. In contrast, those who are diagnosed overt hypothyroidism show an elevated TSH level accompanied by a low free T4 level [3]. Subclinical thyroid disease refers to the situation when increased (hypothyroidism) or decreased (hyperthyroidism) plasma TSH concentration is observed while free T4 and total T3 concentrations are in normal range [4]. Resistance to thyroid hormone (RTH) defines a syndrome in which tissues present reduced sensitivity to the action of TH [5].

The liver plays pleiotropic roles in modulating fatty acid and carbohydrate homeostasis. With abundant energy intake, ATP is preferentially generated through carbohydrate oxidation and glycogen stores are replenished in liver and skeletal muscle. When there is further surplus, the carbohydrate is converted to fatty acids [6], which is referred as de novo lipogenesis (DNL) [7] that gives rise to saturated fatty acids (SFA). Fatty acids also undergo modifications such as elongation and desaturation in liver before they are esterified for storage and secretion. Non-alcoholic fatty liver diseases (NFALD), histologically categorized into non-alcoholic fatty liver (NAFL) and non-alcoholic steatohepatitis (NASH), is a group of diseases characterized by hepatic steatosis without significant alcohol consumption or steatogenic medication [8]. It was reported that there was an enhanced lipogenesis in NAFLD patients [9].

The abnormal TH status is frequently linked to hepatic lipid homeostasis alterations [10]. It was reported that NASH patients had a higher prevalence of hypothyroidism than the control group [11]. Also, the severity of NAFLD is associated with the grade of hypothyroidism in a dose-dependent manner [12]. Moreover, TRβPV/PV mice, a mouse model of RTH due to its impaired TRβ function, developed fatty liver [13]. Hepatic steatosis was observed in a TRα dominant-negative P398H mutation [14]. However, these studies cannot answer the question that whether abnormal TH level directly contributes to the pathogenesis of NAFLD.

Although much has been learnt about the molecular mechanisms of THs regulating gene expression involved in lipogenesis [15], little is known about the effect of disturbed THs on hepatic lipid storage and fatty acid composition. Deranged hepatic fatty acid compositions may reflect disturbed lipogenesis, elongation and desaturation. How TH effects on lipogenesis impact the overall picture of hepatic fatty acid remains to be elucidated in detail.

Since it has been reckoned that TH positively regulates genes involved in hepatic lipogenesis, the use of TR agonists for the treatment of NAFLD has been theoretically considered unviable. However, a bunch of TR agonists have been implicated to potently treat hepatic steatosis [16] or reduce hepatic triglyceride [17]. In addition, the proposed positive regulation of hepatic lipogenesis could not be used to explain the fatty liver developed in TRβPV/PV mice. These suggested that the complex mechanism involved in TH regulating liver lipid homeostasis is still poorly elucidated.

In this study, we aimed to figure out the roles of TH in hepatic lipid homeostasis. To see if the mice with manipulated TH status recapitulated the features observed in NAFLD patient and animal models, we characterized the liver of our mouse model mainly in terms of lipid accumulation. Taking advantage of the gas chromatography-mass spectrometry (GC-MS) methods, we also quantified the hepatic fatty acid composition. At the same time, we determined the expression levels of genes that control the key steps of DNL and fatty acid modification. Collectively, these findings may help to uncover the relationship between NAFLD and disturbed TH level.

## Results

### Liver weight and hepatic lipid content

**Figure 1:**
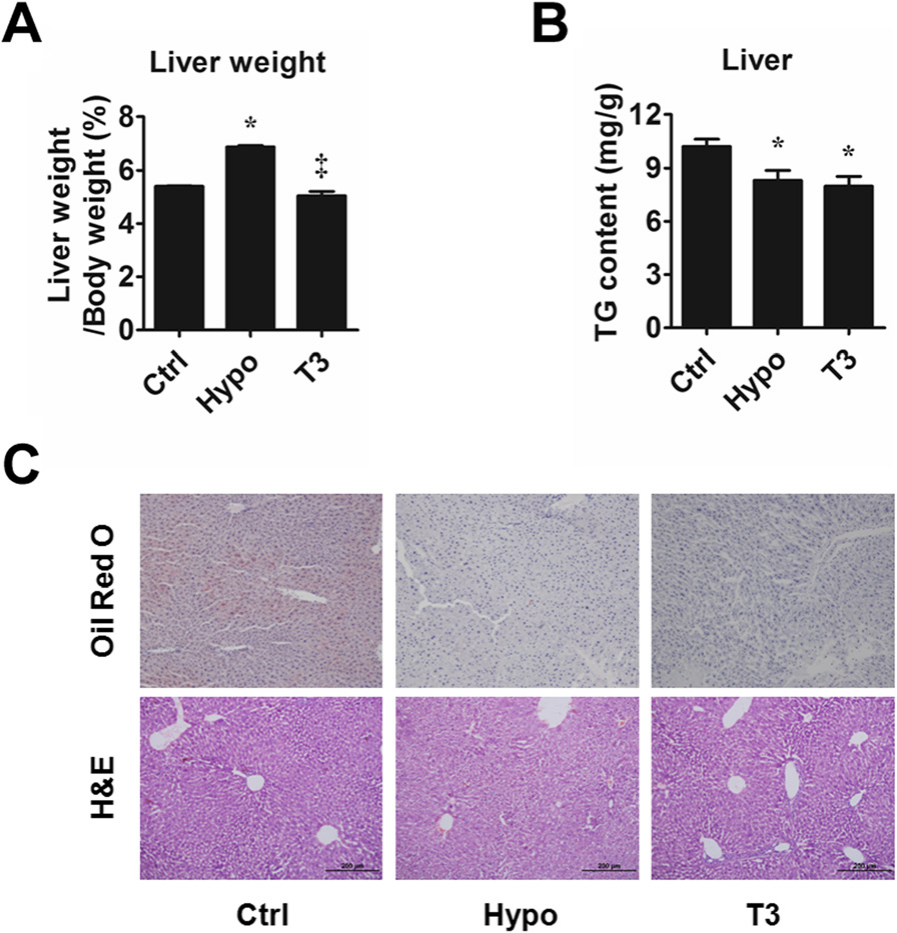
**Liver weight and hepatic lipid content. (A)** Liver weight to body weight ratio of control group (Ctrl), hypothyroid group (Hypo) and T3-treated group (T3) mice. n = 6 in each group. **(B)** Hepatic triglyceride content in Ctrl, Hypo and T3 mice. n = 6 ~ 8 in each group. **(C)** Oil red O staining and H&E staining of Ctrl, Hypo and T3 mice. Scale bar indicates a length of 200μm. *P < 0.05 vs. Ctrl, ^‡^P < 0.05 vs. Hypo.

To investigate whether abnormal TH levels would lead to hepatic features similar to NAFLD, we treated mice with methimazole (MMI) so as to render them hypothyroidism (hypothyroid mice) (Additional file 1: Figure S1) [18]. We found that the MMI treatment led to 27.41% increase in liver-to-body weight ratio in hypothyroid mice compared with control mice (6.88 ± 0.06% v.s. 5.40 ± 0.02%, p < 0.05, n = 5 in each group) (Figure 1A). To find out the cause for the liver weight gain, we first measured the hepatic triglyceride contents to see if steatosis, a hallmark of NAFLD [19], led to liver weight alteration. However, we found that the hepatic triglyceride content was significantly reduced in hypothyroid mice compared with control mice (Figure 1B). Neither did oil-red O staining show hepatic lipid accumulation in hypothyroid mice (Figure 1C). Moreover, H&E staining showed no sign of ballooning in the liver of hypothyroid mice, one of the histological features of NASH (Figure 1C). Collectively, the decreased hepatic lipid accumulation and histological features found in our hypothyroid mice did not recapitulate that of NAFLD.

To find out how TH affects hepatic lipid homeostasis, we treated some mice in hypothyroid group with T3 for 5 days (Additional file 2: Figure S2). We confirmed the effect of T3 treatment by the elevated hepatic type 1 iodothyronine deiodinase (Dio1) level (Additional file 1: Figure S1), a known sensitive marker of peripheral thyroid status [20]. It was observed that T3 treatment resulted in 26.74% decrease in liver-to-body weight ratio compared with that of hypothyroid mice (Figure 1A) (5.04 ± 0.18% v.s. 6.88 ± 0.06%, p < 0.05, n = 5 in each group). In T3-treated mice, the hepatic triglyceride and lipid accumulation were also lower than that of control mice (Figure 1B & C). Besides, no ballooning was observed in T3-treated mice as manifested by H&E staining (Figure 1C). In sum, in our mouse model, the disturbed TH level led to liver weight alteration. Also, hepatic lipid accumulation decreased in hypothyroid and T3-treated mice compared with that of the control.

### Hepatic lipogenesis and saturated fatty acid compositions

**Figure 2:**
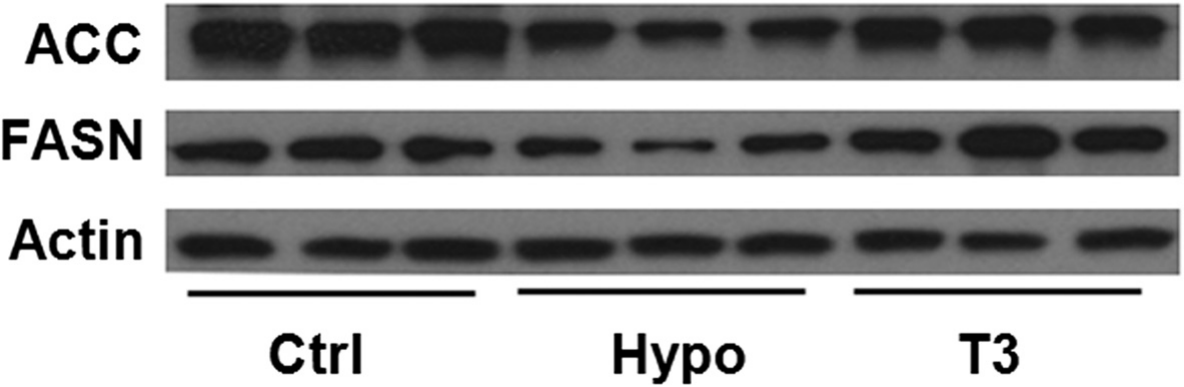
**Hepatic ACC1 and FASN expression levels.** Western blotting analysis of ACC1 and FASN in liver from control group (Ctrl), hypothyroid group (Hypo) and T3-treated group (T3).

Hepatic de novo lipogenesis (DNL) contribute to the lipid content alteration. To investigate if the hepatic DNL was altered under different thyroid hormone status, we compared the expression of two key enzymes involved in DNL, Acetyl-CoA carboxylase 1 (ACC1) and Fatty acid synthase (FASN), between the three groups. It was observed that compared with control mice, ACC1 and FASN decreased on the protein levels in hypothyroid mice; when treated with T3, the protein levels of ACC1 and FASN was restored to a level comparable to the control mice (Figure 2). Compared with the control mice, the mRNA level of ACC1 was increased in hypothyroid mice while it was not significantly reduced in T3-treated mice. As for FASN mRNA, there was no significant difference between control and hypothyroid mice while it was lower in T3-treated mice (Additional file 3: Figure S3). The altered gene expression prompted us to investigate whether the products of DNL in the liver would also be affected by TH status. Since the DNL products belong to the saturated fatty acid (SFA) family, we measured and compared the SFA amount between the three groups (Table 1). The major products of DNL, C16:0 and C18:0 [21,22], decreased significantly in the liver of hypothyroid mice liver compared with control mice (Table 1). The trend among the rest members of SFA was consistent and qualitatively similar across the family, suggesting that the DNL in hypothyroid mice was suppressed. Thus, we proposed that depressed hepatic DNL partly contributed to the decreased lipid accumulation in the liver of hypothyroid mice.

**Table 1:** Liver saturated fatty acid (SFA) quantification from different groups

**Fatty acid**	**Control**	**Hypothyroid**	**T3-treated**
C14:0	57.9 ± 18.4	45.1 ± 11.3	25.5 ± 1.5
C16:0	17093 ± 1256	13271 ± 658*	15322 ± 720^‡^
C18:0	9769 ± 861	7612 ± 424*	12566 ± 1013*^‡^
C20:0	284 ± 27	132 ± 12*	214 ± 18*^‡^
C22:0	254 ± 22	126 ± 10*	374 ± 19*^‡^
C24:0	164 ± 18	139 ± 5	191 ± 17^‡^
**SFA**	27623 ± 1631	21762 ± 937*	28687 ± 1700^‡^

Individual fatty acid amount was measured by GC-MS and normalized to liver weight (nmol/g). Values represent the mean ± S.E. from five mice in the same group.

*P < 0.05 vs. Control group.

^‡^P < 0.05 vs. Hypothyroid group.

On the other hand, in T3-treated mice, it was observed that the levels of the SFA concentration were higher than that of hypothyroid mice (Table 1), which indicated an enhanced hepatic DNL in response to T3 treatment. This was also in consistent with the reduced ACC1 and FASN protein level in hypothyroid mice and increased one in T3-treated group (Figure 2).

### Hepatic unsaturated fatty acid (UFA) compositions and Stearoyl-CoA desaturase (SCD-1)

**Figure 3:**
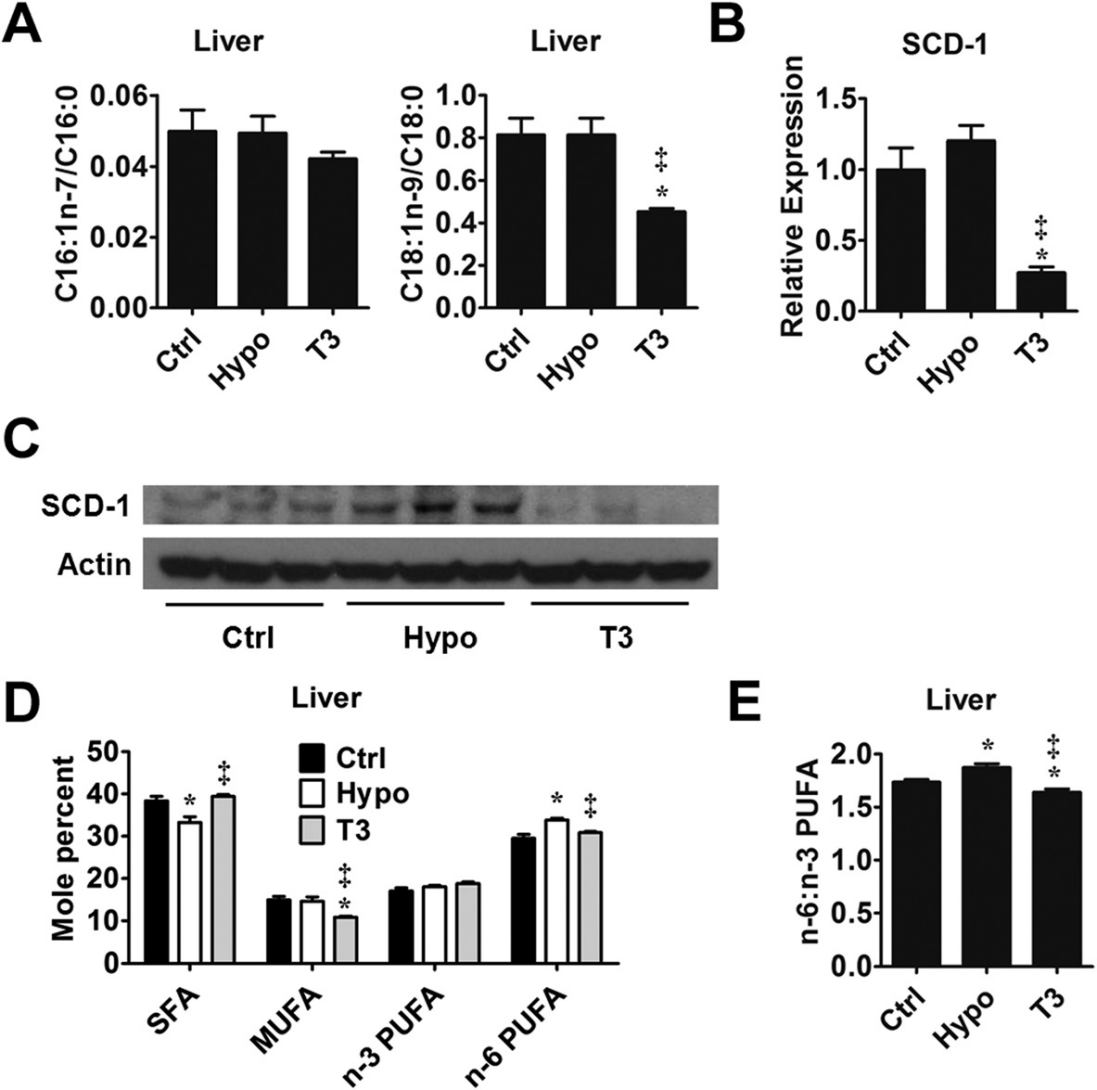
**Unsaturated fatty acid (UFA) concentration and SCD-1 expression. (A)** C16:1n-7/C16:0 and C18:1n-9/C18:0 ratios were compared and used as surrogate measures of hepatic SCD-1 activity. n = 5 in each group **(B)** RT-PCR analysis of SCD-1 gene expression in mouse liver from control group (Ctrl), hypothyroid group (Hypo) and T3-treated group (T3). 18s amplified in parallel served as internal reference. n = 6 in each group. **(C)** Western blotting analysis of SCD-1 with β-Actin as an indication of equal protein loading. **(D)** Fatty acids were grouped according to the degree of saturation and the amount of fatty acid belonging to the same group was added up. The relative contents of each group were compared across the Ctrl, Hypo and T3. n = 5 in each group. **(E)** The ratios between n-6 PUFA and n-3 PUFA in different groups were compared. n = 5 in each group. *P < 0.05 vs. Ctrl, ^‡^P < 0.05 vs. Hypo.

Fatty acids also undergo modifications such as desaturation before they were esterified into triglyceride. Therefore, we also quantified the individual monounsaturated fatty acid (MUFA), the products of desaturation, in the liver from the three groups. As shown in Table 2, the significantly reduced level of C18:1n-9, a most abundant MUFA in the liver, rendered total MUFA concentration lower in T3-treated mice than the control mice. It is known that in the liver, Stearoyl-CoA desaturase (SCD-1) is responsible for the conversion of C16:0 and C18:0 into C16:1n-7 and C18:1n-9, respectively. Thus, we compared the ratios between the products and the precursors and use them as surrogate measures to determine the SCD-1 activity. As shown in Figure 3A, the ratio of C18:1n-9/C18:0 indicated a significantly depressed SCD-1 activity in T3-treated mice compared with the control mice. Although not reaching a statistical significance, the alteration of C16:1n-7/C16:0 showed a similar trend with C18:1n-9/C18:0 (Figure 3A). In agreement with this, we found that the SCD-1 in T3-treated mice was significantly suppressed on the transcriptional and translational level (Figure 3B and C). In hypothyroid mice, we did not find significant difference in SCD-1 activity compared with control mice in regard of the surrogate measures and mRNA levels (Figure 3A & B).

**Table 2:** Liver unsaturated fatty acid (UFA) quantification from different groups

**Fatty acid**	**Control**	**Hypothyroid**	**T3-treated**
C16:1n-7	862 ± 135	660 ± 84	647 ± 46
C16:1n-9	191 ± 22	164 ± 26	95 ± 16*
C18:1n-7	1453 ± 156	1647 ± 228	1209 ± 117
C18:1n-9	7754 ± 547	6789 ± 742	5687 ± 421*
C20:1n-9	282 ± 223	222 ± 26	151 ± 9*
C22:1n-9	88.1 ± 20.6	50.45 ± 13.1	34.0 ± 2.6*
C24:1n-9	144 ± 15	169 ± 10	111 ± 10^‡^
**MUFA**	10774 ± 825	9702 ± 1076	7933 ± 605*
C18:3n-3	231 ± 23	186 ± 21	207 ± 19
C20:5n-3	1174 ± 111	1241 ± 79	2137 ± 187*^‡^
C22:5n-3	653 ± 51	707 ± 56	898 ± 53*^‡^
C22:6n-3	10200 ± 972	9479 ± 421	10362 ± 649
**n-3 PUFA**	12366 ± 1105	11857 ± 571	13722 ± 887
C18:2n-6	13664 ± 970	14302 ± 853	13829 ± 820
C18:3n-6	231 ± 23	186 ± 21	207 ± 19
C20:2n-6	249 ± 23	268 ± 28	210 ± 13
C20:3n-6	1355 ± 125	1807 ± 181	1201 ± 121^‡^
C20:4n-6	5795 ± 646	5630 ± 396	6996 ± 733
C22:4n-6	105 ± 14	93 ± 12	94 ± 11
**n-6 PUFA**	21398 ± 1728	22286 ± 1385	22536 ± 1613
**PUFA**	33764 ± 2822	34144 ± 1952	36258 ± 2485
C18:1t	29.2 ± 6.3	72.9 ± 17.8	25.8 ± 6.1
18:2n-6.9c12t	27.7 ± 1.4	39.0 ± 4.8	34.0 ± 4.5
18:2n-6.9t12c	27.2 ± 6.6	68.6 ± 16.5*	13.2 ± 1.31*^‡^
**TFA**	84.2 ± 9.5	180.5 ± 30.1*	73.0 ± 11.0^‡^

Individual fatty acid amount was measured by GC-MS and normalized to liver weight (nmol/g). Values represent the mean ± S.E. from five mice in the same group.

*P < 0.05 vs. Control group.

^‡^P < 0.05 vs. Hypothyroid group.

As for n-3 and n-6 polyunsaturated fatty acids (PUFAs), we did not observe significant differences between control and hypothyroid mice (Table 2). In the T3-treated mice, hepatic C20:5-3 and C22:5n-3 concentration significantly increased compared with control and hypothyroid mice. C20:3n-6 concentration reduced in T3-treated mice compared with hypothyroid mice (Table 2). Despite the statistically insignificant alteration of C18:1t and C18:2n-6.9c12t, the trans fatty acid (TFA) we detected showed similar patterns of change where the concentration rose in hypothyroid mice and decreased in T3-treated mice (Table 2).

When the amount of fatty acids were added together according to the degree of saturation, it was discovered that SFA level trended downward in hypothyroid mice while in T3-treated mice it was indistinguishable from that of the control mice (Figure 3D). The MUFA content was significantly lower in T3-treated mice than the other two groups (Figure 3D). As is known, both SFA and MUFA are able to be incorporated into triglyceride. However, MUFAs are the more favored the substrates [23,24]. The reduced MUFA level in T3-treated mice might have offset the effect of elevated SFA level in enhancing triglyceride synthesis. Consequently, the triglyceride content was still lower in T3-treated mice compared with the control mice. n-3 PUFA did not show significant alteration among different groups (Figure 3D). On the other hand, n-6 PUFA increased in hypothyroid mice and decreased in T3-treated mice compared with the control (Figure 3D). We also found that compared with the control mice, the n-6/n-3 PUFA ratio significantly elevated in hypothyroid mice and was the lowest in the T3-treated mice (Figure 3E).

### Hepatic glycogen content

**Figure 4:**
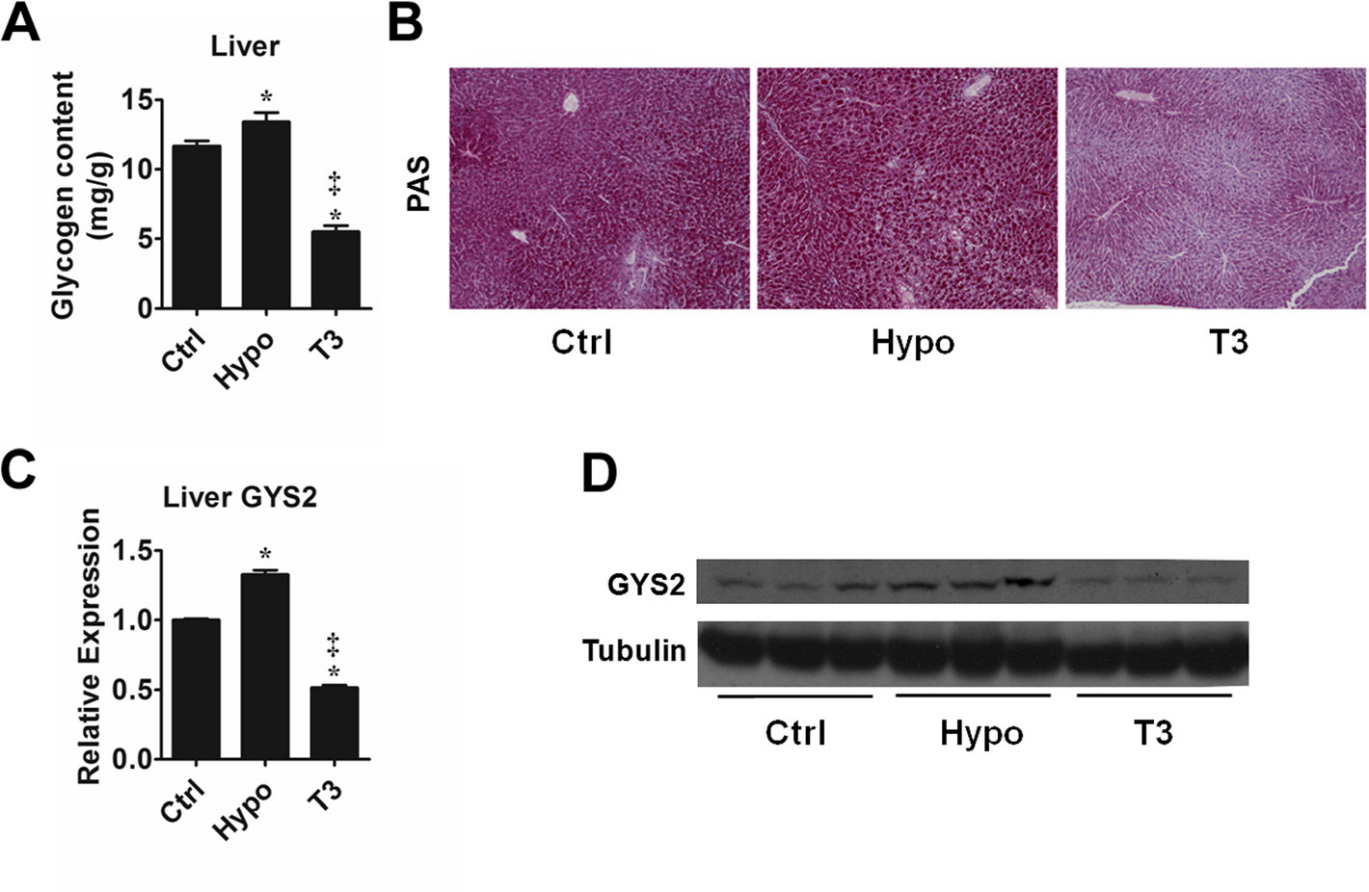
**Hepatic glycogen content and the expression of gene related to glycogen synthesis. (A)** Hepatic glycogen content was measured from liver samples obtained in group control group (Ctrl), hypothyroid group (Hypo) and T3-treated group (T3). n = 6 in each group. **(B)** PAS staining of liver section from Ctrl, Hypo and T3 indicated the presence of glycogen. **(C)** RT-PCR analysis of GYS2 gene expression in mouse liver from Ctrl, Hypo and T3. 18s amplified in parallel served as internal reference. n = 6 ~ 8 in each group. **(D)** Western blotting analysis of GYS2 with α-Tubulin as an indication of equal protein loading. *P < 0.05 vs. Ctrl, ^‡^P < 0.05 vs. Hypo.

Since the triglyceride level could not well explain the liver weight alteration among the groups, we further determined the content of hepatic glycogen, a frequently variable component of liver. It was observed that in hypothyroid mice the glycogen content was higher than the control, whereas in T3-treated mice the glycogen content was lower than both control and hypothyroid mice (Figure 4A). PAS staining displayed a similar result (Figure 4B). In consistency, the expression of liver glycogen synthase (GYS2), the enzyme catalyzing the rate-limiting step in the synthesis of glycogen, increased in hypothyroid mice and decreased in T3-treated mice compared with the control (Figure 4C & D). The inverse relationship between GYS2 expression and thyroid hormone level indicated that it was possible GYS2 expression could be regulated by TH on the transcriptional level. To prove this, luciferase assasy was performed. However, we did not found the fragment we clone from GYS2 promoter responded to T3 (Additional file 4: Figure S4). In sum, we proposed that glycogen content differences might partly account for the liver weight alteration.

## Discussion

T3 is the active form of TH, a well known hormone for its regulation of cellular and tissue metabolism. Through binding to its cognate nuclear receptors with ligand-inducible property, T3 controls a broad range of gene expression in target tissues including liver. The presence of T3 enables TRs to bind to TH response elements in the promoters of target genes and form co-activator complexes to activate transcription [15]. Although studies reported that TH and TRs are potent to control genes involved in lipid and carbohydrate metabolism on transcription level [25,26], they did not necessarily represent the metabolic consequence of the altered TH levels. Our study presents the quantities of major fatty acids within the liver from mice under different TH status. Along with it we also showed that liver glycogen accumulation was affected by TH levels. Both of this provides some novel insights into the picture of liver metabolism under disturbed TH levels.

We did not observe increased triglyceride accumulation, the hallmark of NAFLD, in MMI-induced hypothyroid mice, although the hypothyroid mice showed enlarged livers. In contrast, we found that the main fatty acid products of DNL along with the overall SFA concentration fell significantly in hypothyroid mice, which indicated a depressed fatty acid synthesis in liver. The decreased ACC1 and FASN protein level caused the downward regulated DNL to a great extent. One point worth mentioning is that the mRNA level of ACC1 and FASN in T3-treated mice did not increase. The possible reason was that in our research, T3-treated mice were euthanized 24h later after the last T3 injection, at which their serum T3 level was not significantly different from that of control mice (Additional file 1: Figure S1A) due to the short half life of serum T3 [20]. As a result, the ACC1 and FASN transcription was not elevated in T3-treated group compared with the control. The lack of triglyceride accumulation in our hypothyroid mouse model suggested that hypothyroidism caused by MMI may not cause the steatosis in NAFLD. We could not rule out the possibility that MMI-induced hypothyroidism model and clinically diagnosed hypothyroidism are different. Thus, population based cohort studies are required to confirm our result.

**Figure 5:**
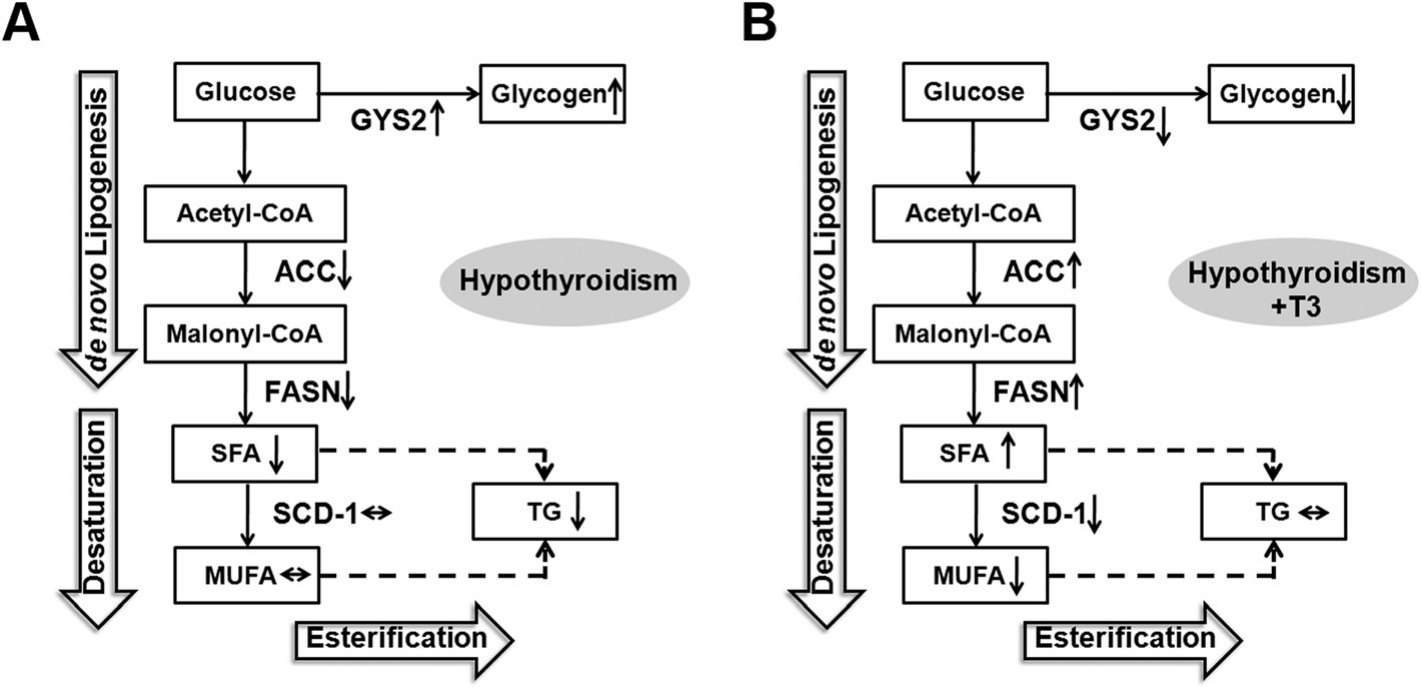
**Flow charts illustrating the preferred metabolic pathways under hypothyroid status before and after T3 treatment.** The hypothyroidism was compared with Control **(A)** while hypothyroidism + T3 status was compared with hypothyroidism **(B)**.

When comparing the T3-treated mice to the hypothyroid mice, we observed elevated DNL product accumulation as well as gene expression. However, no clue indicated enhanced triglyceride accumulation in the T3-treated mice. The main reason for this was that the depressed SCD-1 function led to decreased MUFA, the other critical component of triglyceride (Figure 5). The lack of triglyceride accumulation in T3-treated mice also indicated the possibility of an enhanced fatty acid oxidation which overriding the effect of DNL. It has been proved in various studies that T3 can positively affect genes involved in fatty acid oxidation [27,28] while in hypothyroidism fatty acid oxidation is greatly decelerated. To be noted, the three TFA we detected mainly came from food ingested [29]. Since the food maintained constant gradient throughout experiment, the altered TFA level could be partly ascribed to its oxidation rate. This enables us to propose that the elevated TFA concentration observed in hypothyroid mice could be resulted from decreased rates of fatty acid oxidation (Table 2). Indeed, T3 treatment for 5 days significantly decreased the total TFA concentration compared with hypothyroid mice, indicating enhanced fatty acid oxidation in the T3-treated mice.

Our study further corroborates that SCD-1, the main enzyme responsible of the conversion of SFA to MUFA, was negatively regulated by T3 [30] and [31]. Mice lack SCD-1 exhibited enhanced beta-oxidation [32]. *In vivo* evidence supported that decreased activity of SCD-1 was linked to favourable outcome of liver in terms of lipid accumulation and secretion. However, there was also study showing that lack of SCD-1 activity would sensitize hepatocyte to SFA-induced apoptosis and render mice lack SCD-1 vulnerable to liver damage other than steatosis [23]. The evidence collectively manifests the importance of relative composition of fatty acids in a defined pool.

The untoward effect like extra hepatic effect greatly impeded T3 application to treat NAFLD. In view of this dilemma, a number of TR agonists with either isoform selectivity or tissue specificity have been developed to lower hepatic triglyceride levels [16,17,33]. It is worthwhile to study the effect of TR agonists in modulating DNL and SCD-1 activity so as to set better therapeutic windows.

Similar to the liver in the NAFLD [34], we also observed an increased hepatic n-6/n-3 PUFA ratio in hypothyroid mice, although in a different pool of lipid. In response to T3 treatment, the ratio decreased to a level even lower than the control mice. Plenty efforts have been spared in addressing what the PUFA ratio indicates. But most of them focused on the ratios in different dietary sources. It still deserves additional research to uncover the indication of increased hepatic n-6/n-3 PUFA ratio.

Another point noteworthy is that hypothyroidism and T3 treatment affect glycogen accumulation, an important aspect of energy metabolism. The clinical relevance of hepatic glycogen content and thyroid disease is not well studied. Previous studies did not uncover the effect of abnormal TH level on the hepatic glycogen accumulation [35,36]. Our data showed that glycogen accumulation increased in hypothyroid mice whereas with T3 treatment it decreased significantly. Glycogen is the most instant energy source stored in liver. As noted, DNL was suppressed in hypothyroid mice. This suppression could direct the ingested surplus carbohydrate to store in the form of glycogen (Figure 5). The overall alterations led to an elevated level of hepatic glycogen and declining triglyceride and lipid accumulation. After all, the weight gain and loss in the liver might be partly ascribed to glycogen amount.

## Conclusions

In conclusion, our study provides several novel observations about the alterations in the hepatic fatty acid composition and glycogen storage in mice under disturbed TH status (Figure 5). These observations may provide novel insight into the relationship between TH and liver metabolism. The results can be referred to improve our knowledge concerning the mechanism involved in TH regulating liver lipid and carbohydrate homeostasis. Moreover, this might be helpful in advising the development of TH analogues to treat NAFLD and related diseases such as metabolism syndrome.

## Methods

### Animals

Animals were maintained and experiments were performed according to protocols approved by the Animal Care and Use Committees of Institute for Nutritional Sciences. 10 to12-week old male C57BL/6 mice were used in all cases. Mice were rendered hypothyroid by the addition of 0.1% methimazole (MMI) and 1% NaClO_4_ (Sigma, St. Louis, MO) in their drinking water for 28 days [37]. As indicated, some animals from this group were injected intraperitoneally with 5µg T3/20 g body weight per day for 5 days, on day 23–27 of MMI/NaClO_4_ treatment. Mice were euthanized on the 28th day of MMI treatment or 24 h after their last T3 injection by exsanguination under anesthesia on day 28. Control mice received no treatment [18]. The treatment was also illustrated schematically in Additional file 4: Figure S4. We evaluated the serum T3 and hepatic Dio1 transcript levels confirmed the establishment of intended hypothyroid status in MMI-treated mice (Additional file 1: Figure S1A and B).

### Liver sample collection, fatty acid extraction and FAME preparation

Mice were euthanized under anesthesia. The dissected liver was cut into several small pieces and immediately frozen in liquid nitrogen or fixed in polyformalin for histological analysis. The liver, weighing 14-18 mg, was first homogenized in ice cold PBS added with internal standard. Fatty acids were extracted by hexane and isopropanol and then incubated with a mixture of methanol and sulfuric acid to produce fatty acid methyl esters (FAMEs). FAMEs were separated by gas chromatography (Agilent 6890 GC; SP-2560 capillary column: 100 m × 0.25 mm internal diameter × 0.2 μm film; Supelco). Individual FAMEs were identified by positive chemical ionization with the use of methane as the reagent gas (Agilent 5975B) [38,39].

### Real-time RT-PCR and western blot analysis

Total RNA was isolated by using TRIzol reagent (Invitrogen, USA) according to the manufacturer’s instructions. The isolated total RNA was reverse-transcribed by using PrimeScript RT reagent Kit (TaKaRa, Shiga, Japan). Real-time PCR was performed on an ABI 7900 Real-Time PCR System (Applied Biosystems). Specific primers for Real-time PCR were listed in Additional file 5: Table S1. The gene expression is quantified by normalizing to 18s.

Western Blot analysis was performed as described before [40]. Anti-SCD-1 (Santa Cruz Biotechnology, Santa Cruz, CA), anti-FASN (BD, Biosciences PharMingen, San Diego, CA, USA), anti-ACC1 (Cell Signaling Technology, Danvers, MA), anti-β-Actin (Sigma), anti-α-Tubulin (Sigma), and anti-GYS2 (Cell Signaling Technology) antibodies were used as primary antibodies.

### Histological analysis

To stain the neutral lipid, liver specimens were frozen in liquid nitrogen and cryostat sections were used for Oil Red O staining. For immunohistochemical staining, liver specimens were fixed in 10% neutral buffered formalin and subsequently embedded in paraffin for section. Periodic acid-Schiff (PAS) staining was used to indicate the liver glycogen.

### Triglyceride content, glycogen content and serum T3 level determination

Hepatic triglyceride content was determined by Wako triglyceride lab assay kit (Wako Pure Chemical Industries, Osaka, Japan). Hepatic glycogen content was measured by Glycogen assay kit (Keygen Biotech, Nanjing, China). Blood samples were centrifuged to prepare serum and stored at −80°C. Serum total T3 concentrations were determined in Ruijin Hospital affiliated to Shanghai Jiaotong University.

### Plasmid, transfection and luciferase assay

For construction of the luciferase reporter plasmid, the ~1.2 kbps promoter region of glycogen synthase 2 was amplified from mouse DNA by PCR and inserted into the pGL3-basic vector, which was designated as GYS2-luc. Transfection was performed using Lipofectamine 2000 (Invitrogen, Carlsbad, CA, USA). GYS2-luc or Pal-luc was co-transfected with TRα or TRβ expression plasmid. 12 h later, T3 was added at indicated dose. Luciferase assays were performed by using the Dual-Luciferase Reporter Assay System (Promega, Madison, WI, USA) as described previously [18]. Luciferase activity was measured on a luminometer (Berthold Technologies, Bad Wildbad, Germany).

### Calculations and statistics

Summary data for the fatty acid classes, their relative quantities to liver weight percent and mole percent (percentage of each fatty acid of the total fatty acids we determined by GC-MS) were calculated. The results were expressed as means ± SEM. A nonparametric Wilcoxon signed-rank test was used for two-group comparison. When three groups were compared, analysis of variance was used. A *P* value of 0.05 or less was regarded as statistically significant.

## Abbreviations

ACC1: Acetyl-CoA carboxylase 1; Dio1: Type 1 iodothyronine deiodinase; DNL: De novo lipogenesis; FAMEs: Fatty acid methyl esters; FASN: Fatty acid synthase; GC-MS: Gas chromatography–mass spectrometry; GYS2: Glycogen synthase; MMI: Methimazole; MUFA: Mono unsaturated fatty acid; NAFL: Non-alcoholic fatty liver; NASH: Non-alcoholic steatohepatitis; NFALD: Non-alcoholic fatty liver diseases; PAS: Periodic acid-Schiff; PUFA: Poly unsaturated fatty acid; RTH: Resistance to thyroid hormone; RT: Reverse transcription; SCD-1: Stearoyl-CoA desaturase-1; SFA: Saturated fatty acids; TFA: Trans fatty acid; TH: Thyroid hormone; T4: Thyroxine; T3: Triiodothyronine; TSH: Thyroid-stimulating hormone; TR: TH receptors; UFA: Unsaturated fatty acid.

## Competing interests

The authors declare that they have no competing interests.

## Authors’ contributions

XY carried out the studies, participated in the animal experiment and FAME preparation, and drafted the manuscript. SH participated in the FAME preparation and GC-MS data extraction. DZ and HX participated in the animal experiment. YCW, HY and JJ participated in the design of the study. HY conceived of the study, and participated in its design and coordination and helped to draft the manuscript. All authors read and approved the final manuscript.

## Supplementary Material

Additional file 1: Figure S1 Serum T3 levels and hepatic type 1 iodothyronine deiodinase (Dio1) mRNA determination. (A) Serum T3 levels were determined in control group (Ctrl), hypothyroid group (Hypo) and T3-treated group (T3). n = 5 in each group. Error bars represent the SEM. The serum T3 level of Hypo mice was below 0.25 ng/ml, the lower limit of detection. (B) RT-PCR analysis of Dio1 gene expression in mouse livers from control group (Ctrl), hypothyroid group (Hypo) and T3-treated group (T3). 18s amplified in parallel served as internal reference. n = 6 ~ 8 in each group. *P < 0.05 vs. Ctrl, ^‡^P < 0.05 vs. Hypo. Click here for file

Additional file 2: Figure S2 Schematic of different treatments and designation of groups. Mice of indicated age were treated with 0.1% MMI and 1% NaClO4 in their drinking water for a total of 28 days (hypothyroid), not treated (control), or treated with 0.1% MMI and 1% NaClO4 in their drinking water for 28 days and injected with 5μg T3/20 g body weight per day at 24-h intervals on day 23–27 (T3-treated). Twenty-four hours after the last T3 injection, on day 28, mice were euthanized by exsanguination, and serum and tissues were collected. Click here for file

Additional file 3: Figure S3 Hepatic ACC1 and FASN mRNA levels. RT-PCR analysis of ACC1 and FASN gene expression in mouse livers from control group (Ctrl), hypothyroid group (Hypo) and T3-treated group (T3). 18s amplified in parallel served as internal reference. n = 6 ~ 8 in each group. *P < 0.05 vs. Ctrl, ^‡^P < 0.05 vs. Hypo. Click here for file

Additional file 4: Figure S4 Determination of TR effect on the promoter activity of GYS2. 293T were cotransfected with a reporter containing GYS2 promoter (GYS2-luc) or positive control of thyroid hormone respone element (pal-luc), and TRα or TRβ expression plasmids as indicated. 12 hours later, 100nM T3 was added into the medium as indicated. Luciferase activity was determined 24 hours after T3 was added. Error bars represent the SEM of three independent experiments. ***P < 0.05 Ctrl vs T3 (100 nM). Click here for file

Additional file 5: Table S1 Specific primers for Real-time PCR. Click here for file
