# Supplementary figures and images for: Regulation of fatty acid composition and lipid storage by thyroid hormone in mouse liver
Source: Cell Biosci. 2014 Jul 30;4:38. doi: 10.1186/2045-3701-4-38 (PMC4124172; doi:10.1186/2045-3701-4-38)

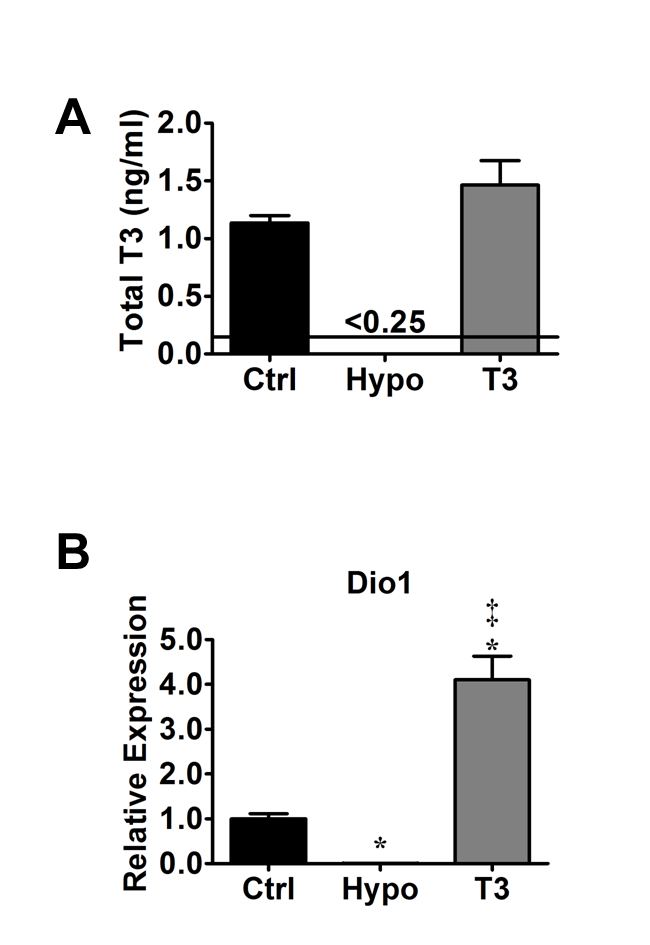

Supplement: Additional file 1: Figure S1 — Serum T3 levels and hepatic type 1 iodothyronine deiodinase (Dio1) mRNA determination. (A) Serum T3 levels were determined in control group (Ctrl), hypothyroid group (Hypo) and T3-treated group (T3). n = 5 in each group. Error bars represent the SEM. The serum T3 level of Hypo mice was below 0.25 ng/ml, the lower limit of detection. (B) RT-PCR analysis of Dio1 gene expression in mouse livers from control group (Ctrl), hypothyroid group (Hypo) and T3-treated group (T3). 18s amplified in parallel served as internal reference. n = 6 ~ 8 in each group. *P < 0.05 vs. Ctrl, ‡P < 0.05 vs. Hypo. [file 2045-3701-4-38-S1.tiff]

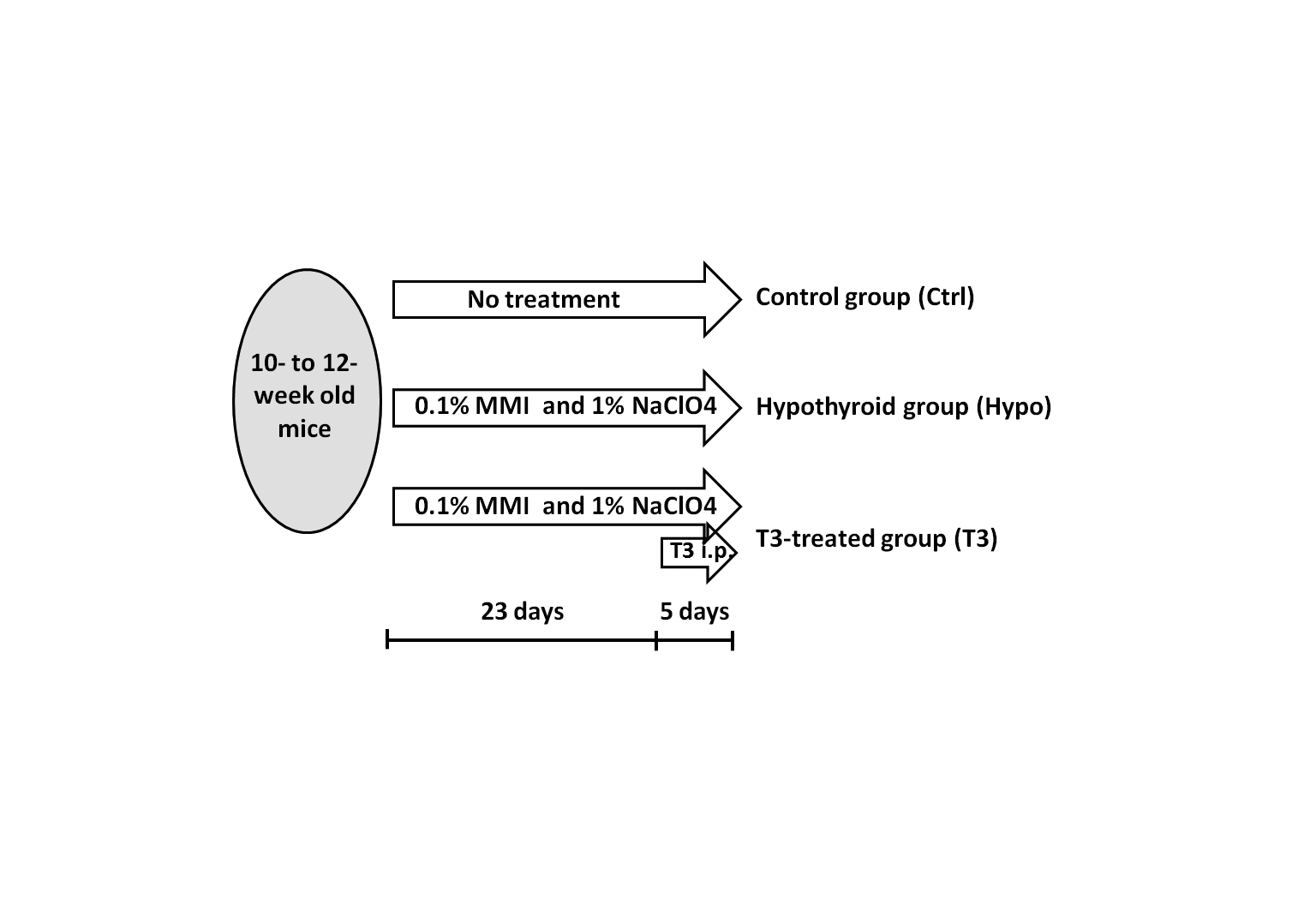

Supplement: Additional file 2: Figure S2 — Schematic of different treatments and designation of groups. Mice of indicated age were treated with 0.1% MMI and 1% NaClO4 in their drinking water for a total of 28 days (hypothyroid), not treated (control), or treated with 0.1% MMI and 1% NaClO4 in their drinking water for 28 days and injected with 5μg T3/20 g body weight per day at 24-h intervals on day 23–27 (T3-treated). Twenty-four hours after the last T3 injection, on day 28, mice were euthanized by exsanguination, and serum and tissues were collected. [file 2045-3701-4-38-S2.tiff]

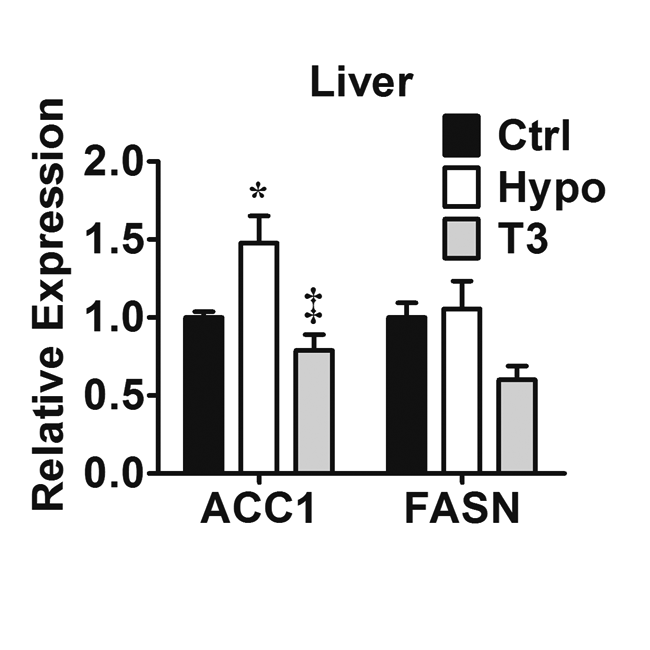

Supplement: Additional file 3: Figure S3 — Hepatic ACC1 and FASN mRNA levels. RT-PCR analysis of ACC1 and FASN gene expression in mouse livers from control group (Ctrl), hypothyroid group (Hypo) and T3-treated group (T3). 18s amplified in parallel served as internal reference. n = 6 ~ 8 in each group. *P < 0.05 vs. Ctrl, ‡P < 0.05 vs. Hypo. [file 2045-3701-4-38-S3.tiff]

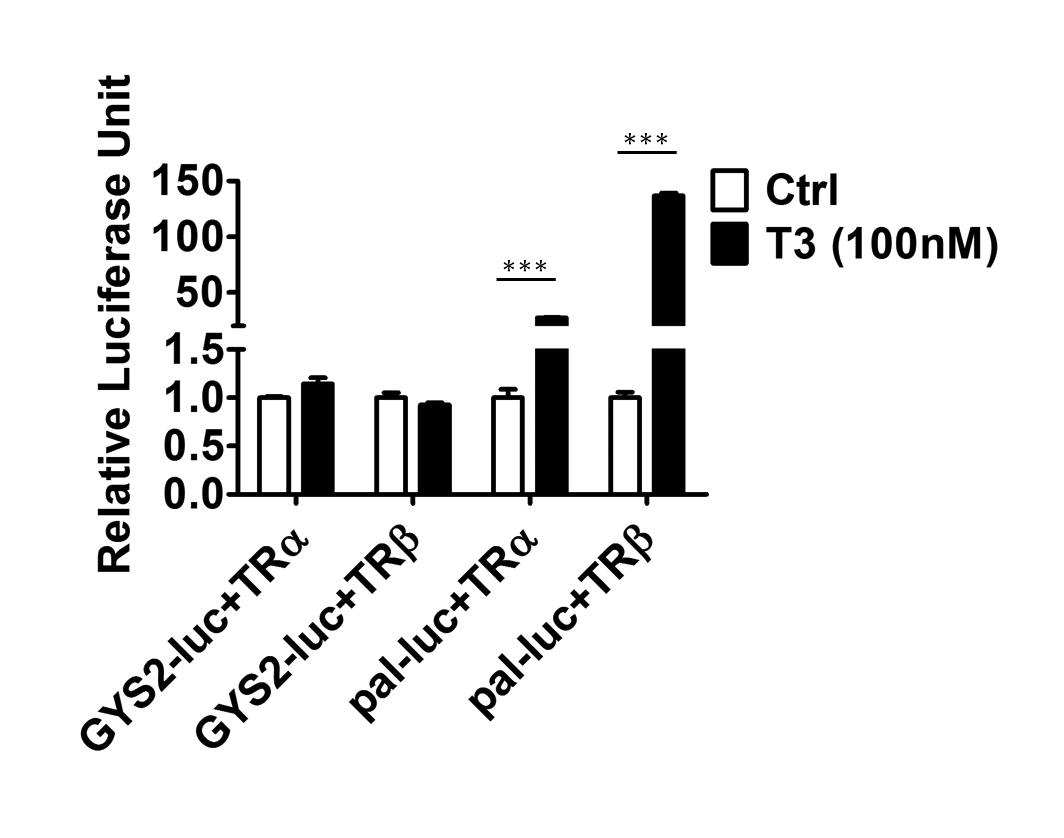

Supplement: Additional file 4: Figure S4 — Determination of TR effect on the promoter activity of GYS2. 293T were cotransfected with a reporter containing GYS2 promoter (GYS2-luc) or positive control of thyroid hormone respone element (pal-luc), and TRα or TRβ expression plasmids as indicated. 12 hours later, 100nM T3 was added into the medium as indicated. Luciferase activity was determined 24 hours after T3 was added. Error bars represent the SEM of three independent experiments. ***P < 0.05 Ctrl vs T3 (100 nM). [file 2045-3701-4-38-S4.tiff]
