# Supplementary material for: Regulation of fatty acid composition and lipid storage by thyroid hormone in mouse liver
Source: Cell Biosci. 2014 Jul 30;4:38. doi: 10.1186/2045-3701-4-38 (PMC4124172; doi:10.1186/2045-3701-4-38)
Supplement: Additional file 5: Table S1 — Specific primers for Real-time PCR. [file 2045-3701-4-38-S5.docx]

## Additional Table S1. Specific primers for Real-time PCR

| Gene |  | Sequence |
| --- | --- | --- |
| ACC1 | sense | CGCCAACAATGGTATTGCAG |
|  | antisense | ACTCCACATTTGCGTAATTGTTG |
| FASN | sense | GGAGGTGGTGATAGCCGGTAT |
|  | antisense | TGGGTAATCCATAGAGCCCAG |
| SCD-1 | sense | TTCTTGCGATACACTCTGGTGC |
|  | antisense | CGGGATTGAATGTTCTTGTCGT |
| GYS2 | sense | GACCAAGGCCAAAACGACAG |
|  | antisense | AGAGCACCACGTAGGGACT |
| Dio1 | sense | CCACCTTCTTCAGCATCC |
|  | antisense | AGTCATCTACGAGTCTCTTG |
| 18s | sense | ACCGCAGCTAGGAATAATGGA |
|  | antisense | CAAATGCTTTCGCTCTGGTC |
